# DNA methylation profiling in the Carolina Breast Cancer Study defines cancer subclasses differing in clinicopathologic characteristics and survival

**DOI:** 10.1186/s13058-014-0450-6

**Published:** 2014-10-07

**Authors:** Kathleen Conway, Sharon N Edmiston, Ryan May, Pei Fen Kuan, Haitao Chu, Christopher Bryant, Chiu-Kit Tse, Theresa Swift-Scanlan, Joseph Geradts, Melissa A Troester, Robert C Millikan

**Affiliations:** 1 Department of Epidemiology, School of Public Health, University of North Carolina, Campus Box 7435, Chapel Hill, 27599 NC USA; 2 Lineberger Comprehensive Cancer Center, University of North Carolina, Chapel Hill, 27599 NC USA; 3 The EMMES Corporation, Rockville, 20850 MD USA; 4 Department of Applied Mathematics and Statistics, Stony Brook University, Stony Brook, 11794-3600 NY USA; 5 Division of Biostatistics, MMC 303 School of Public Health, University of Minnesota, Minneapolis, 55455 MN USA; 6 Department of Biostatistics, School of Public Health, University of North Carolina, Chapel Hill, 27599 NC USA; 7 School of Nursing, University of North Carolina, Chapel Hill, Campus Box 7460, 27599 NC USA; 8 Department of Pathology, School of Medicine, Duke University Medical Center DUMC 3712, Durham, 27710 NC USA

## Abstract

**Introduction:**

Breast cancer is a heterogeneous disease, with several intrinsic subtypes differing by hormone receptor (HR) status, molecular profiles, and prognosis. However, the role of DNA methylation in breast cancer development and progression and its relationship with the intrinsic tumor subtypes are not fully understood.

**Methods:**

A microarray targeting promoters of cancer-related genes was used to evaluate DNA methylation at 935 CpG sites in 517 breast tumors from the Carolina Breast Cancer Study, a population-based study of invasive breast cancer.

**Results:**

Consensus clustering using methylation (β) values for the 167 most variant CpG loci defined four clusters differing most distinctly in HR status, intrinsic subtype (luminal versus basal-like), and p53 mutation status. Supervised analyses for HR status, subtype, and p53 status identified 266 differentially methylated CpG loci with considerable overlap. Genes relatively hypermethylated in HR^+^, luminal A, or p53 wild-type breast cancers included *FABP3*, *FGF2*, *FZD9*, *GAS7*, *HDAC9*, *HOXA11*, *MME*, *PAX6*, *POMC*, *PTGS2*, *RASSF1*, *RBP1*, and *SCGB3A1*, whereas those more highly methylated in HR-, basal-like, or p53 mutant tumors included *BCR*, *C4B*, *DAB2IP*, *MEST*, *RARA*, *SEPT5*, *TFF1*, *THY1*, and *SERPINA5.* Clustering also defined a hypermethylated luminal-enriched tumor cluster 3 that gene ontology analysis revealed to be enriched for homeobox and other developmental genes (*ASCL2*, *DLK1*, *EYA4*, *GAS7*, *HOXA5*, *HOXA9*, *HOXB13*, *IHH*, *IPF1*, *ISL1*, *PAX6*, *TBX1*, *SOX1*, and *SOX17*). Although basal-enriched cluster 2 showed worse short-term survival, the luminal-enriched cluster 3 showed worse long-term survival but was not independently prognostic in multivariate Cox proportional hazard analysis, likely due to the mostly early stage cases in this dataset.

**Conclusions:**

This study demonstrates that epigenetic patterns are strongly associated with HR status, subtype, and p53 mutation status and may show heterogeneity within tumor subclass. Among HR^+^ breast tumors, a subset exhibiting a gene signature characterized by hypermethylation of developmental genes and poorer clinicopathologic features may have prognostic value and requires further study. Genes differentially methylated between clinically important tumor subsets have roles in differentiation, development, and tumor growth and may be critical to establishing and maintaining tumor phenotypes and clinical outcomes.

**Electronic supplementary material:**

The online version of this article (doi:10.1186/s13058-014-0450-6) contains supplementary material, which is available to authorized users.

## Introduction

Breast cancer is a complex and heterogeneous disease composed of several major subtypes with different molecular alterations, clinical behavior, and outcomes [1]-[3]. Microarray-based gene expression profiling of breast tumors has identified at least six major intrinsic subtypes—luminal A, luminal B, human epidermal growth factor receptor 2-positive/estrogen receptor-negative (HER2^+^/ER^−^), basal-like, claudin-low, and normal-like—that are thought to originate from different precursor cells and follow different progression pathways [4]-[6]. In addition, the genetic pathways leading to breast cancer vary by subtype. For example, basal-like tumors exhibit the highest and the luminal A tumors exhibit the lowest prevalence of p53 mutations [7]. These intrinsic subtypes differ in incidence by race and menopausal status [7] and show differences in risk factors [8], outcomes [7],[9], and responsiveness to chemotherapy [10].

Although genetic alterations such as mutations, rearrangements, and copy number changes are established contributors to carcinogenesis, epigenetic alterations, including DNA methylation, also play an integral role. DNA methylation most commonly occurs when a methyl group is added to a cytosine preceding a guanosine (CpG). CpGs are often found at high densities in `CpG islands', particularly within the promoter regions of genes; hypermethylation of CpG islands can result in the transcriptional silencing of tumor suppressor genes in cancer, whereas CpG hypomethylation may lead to gene activation [11]-[13]. Because alterations in DNA methylation often occur early in cancer development, candidate methylation markers may be valuable for early, specific cancer detection or for predicting clinical response to therapeutic agents or cancer prognosis.

In this study, we characterized DNA methylation profiles by using a microarray approach targeting CpG loci in the promoters of cancer-related genes in 517 breast tumors from the Carolina Breast Cancer Study (CBCS), a large, population-based study of mostly early-stage breast cancer in North Carolina. We hypothesized that DNA methylation events might be important determinants of tumor biology, could delineate tumor groups with distinct survival differences, and may help identify early etiologic events in breast carcinogenesis. In this report, we describe the results of this DNA methylation profiling analysis, focusing on the identification of tumor subclasses and the differentially methylated genes distinguishing them, survival differences among these tumor clusters, and characterization of hypermethylated breast tumors that may be manifestations of the CpG island methylator phenotype (CIMP) originally observed in colorectal cancer [14].

## Materials and methods

### Carolina Breast Cancer Study population

The CBCS is a population-based, case-control study of breast cancer. Participants include women, 20 to 74 years old, residing in 24 contiguous counties of central and eastern North Carolina [15]. Women with a first diagnosis of invasive breast cancer between 1993 and 1996 (phase 1 of the CBCS) were identified by the North Carolina Central Cancer Registry through a rapid case ascertainment system. Women diagnosed prior to age 50 and African-American women were over-sampled to ensure that they comprised roughly half the study sample. Race was self-reported; additional details are included in Table 1. Additional details of the study design are described elsewhere [15],[16]. This study was approved by the Institutional Review Board at the University of North Carolina (UNC) School of Medicine. In total, 861 breast cancer cases were eligible for and consented to participate in the CBCS during phase 1. All CBCS patients provided written informed consent. Epidemiologic risk factor information was obtained from questionnaires that were administered to participants in their homes by trained nurse-interviewers. Clinical data and information on tumor characteristics were obtained from medical records or direct histopathologic review of tumor tissue. ER and progesterone receptor (PR) status of breast tumors was determined primarily through review of medical records (90% of cases) and by immunohistochemistry (IHC) staining in the remaining cases in the Tissue Procurement and Analysis Facility at UNC as described previously [7].

**Table 1:** **Characteristics of Carolina Breast Cancer Study breast cancer cases or tumors evaluated or not evaluated for DNA methylation profile**

Characteristic		Cases evaluated for methylation		Cases not evaluated		*P*value
		(n =517)		(n =163)		
		N	(%)	N	(%)	
Age	Mean ± SD, years	49.7 ± 11.9		52.2 ± 11.9		
	<50 years	318	(62)	84	(52)	0.02
	50+ years	199	(38)	78	(48)	
Race						
	White/Other^a^	301	(58)	106	(65)	0.12
	African-American	216	(42)	57	(35)	
Menopausal status						
	Premenopausal	275	(53)	76	(47)	0.14
	Postmenopausal	242	(47)	87	(53)	
Stage^b^						
	I	178	(37)	68	(45)	0.37
	II	245	(51)	67	(44)	
	III	45	(9)	12	(8)	
	IV	13	(3)	4	(3)	
Primary tumor size						
	≤2 cm	250	(50)	87	(57)	0.28
	>2-5 cm	205	(41)	52	(34)	
	>5 cm	42	(9)	13	(9)	
Lymph node status						
	Negative	291	(58)	101	(66)	0.11
	Positive	207	(42)	53	(34)	
Hormone receptor expression						
	ER^+^/PR^+^	250	(50)	62	(42)	0.38
	ER^+^/PR^-^	48	(10)	17	(12)	
	ER^-^/PR^+^	39	(8)	15	(10)	
	ER^-^/PR^-^	163	(33)	53	(36)	
Combined tumor grade^c^						
	I	126	(25)	47	(29)	0.48
	II	156	(30)	49	(30)	
	II	228	(45)	65	(40)	
Histologic type						
	Ductal^d^	388	(75)	122	(75)	0.23
	Ductal variants^e^	13	(3)	9	(5)	
	Poorly differentiated^f^	22	(4)	3	(2)	
	Lobular^g^	46	(9)	13	(8)	
	Mixed lobular/Ductal	48	(9)	16	(10)	
IHC intrinsic subtype^h^						
	Luminal A	212	(51)	42	(51)	0.76
	Luminal B	65	(16)	12	(15)	
	Basal-like	86	(21)	14	(17)	
	HER2^+^/HR^-^	26	(6)	7	(8)	
	Unclassified	24	(6)	7	(8)	
p53 mutation status						
	Positive	218	(42)	47	(34)	0.08
	Negative	297	(58)	91	(66)	

^a^The white/other cases evaluated included 291 Caucasians, 3 American Indians, 6 Asians, and 1 other. ^b^According to the American Joint Committee on Cancer breast tumor staging guidelines. ^c^Nottingham grade based on mitotic index, histologic grade, and nuclear grade. ^d^Ductal not otherwise specified (n = 372), medullary (n = 3), neuroendocrine (n =2), apocrine (n = 2), and other mixed (n = 9). ^e^Ductal variants include mucinous (n =8), papillary (n = 1), and cribriform (n = 4). ^f^Poorly differentiated include metaplastic carcinoma (n = 6), anaplastic carcinoma (n =3), and undifferentiated high grade carcinoma (n = 13). ^g^Lobular, classic, and/or variant (n = 46). ^h^Intrinsic subtype was determined by estrogen receptor (ER), progesterone receptor (PR), and human epidermal growth factor receptor 2 (HER2) status determined by medical records or immunohistochemistry (IHC), and IHC staining for CK5, CK6, and epidermal growth factor receptor. HR, hormone receptor; SD, standard deviation.

### Tumor tissue preparation and histopathologic evaluation

Formalin-fixed paraffin-embedded (FFPE) tumor blocks were obtained from pathology departments at participating hospitals for 798 of the 861 breast cancer cases eligible for phase 1 of the CBCS. Of these, 684 had sufficient tumor tissue for molecular analyses. Tumors were sectioned as previously described [17] and underwent standardized histopathologic review by the study pathologist (JG) to confirm diagnosis, determine histologic subtype, and score standard histopathology features (grade, mitotic index, and so on). With the hematoxylin-and-eosin-stained slide used as a guide, the area of invasive tumor was selectively dissected away from other surrounding non-tumor tissue and then processed for DNA.

### Breast tumor intrinsic subtypes

Subtypes were previously identified [7] by using a panel of IHC protein markers to assess expression of ER, PR, HER2, cytokeratins 5 and 6 (CK5 and CK6), and epidermal growth factor receptor (EGFR). Subtypes included luminal A (HR^+^ (ER^+^ or PR^+^ or both) and HER2^-^), luminal B (HR^+^/HER2^+^), basal-like (HR^-^/HER2^-^/CK5^+^ or CK6^+^ or both), HER2^+^/HR^-^, and unclassified (all markers negative). This IHC marker panel was previously validated against gene expression profiles [18] and was found to provide superior classification of basal-like tumors and outcome prediction over the triple-negative markers [19].

### Normal breast tissues

Nine FFPE histologically normal breast tissues from women without cancer or other premalignant breast conditions were obtained from the Tissue Procurement Facility at UNC and processed for DNA. Patient consent was provided to the facility and tissues were dispersed to this study in anonymized form.

### DNA extraction

FFPE tissues were processed for DNA lysates by using a Proteinase K extraction method as previously described [20].

### p53 mutation screening

P53 mutation screening of 656 FFPE breast tumors in the CBCS was previously accomplished by using a combination of the Roche p53 AmpliChip (Roche Molecular Systems, Pleasanton, CA, USA), single-strand conformational polymorphism analysis, and direct radio-labeled DNA sequencing. Details of the p53 methods are provided in Additional file 1.

### Bisulfite treatment of DNA

Sodium bisulfite modification of DNA obtained from FFPE tissue was performed by using the EZ DNA Methylation Gold kit (Zymo Research, Orange, CA, USA) as previously described [21].

### Illumina GoldenGate Cancer Panel I methylation array analysis

Array-based DNA methylation profiling was accomplished by using the Illumina GoldenGate Cancer Panel I methylation bead array to simultaneously interrogate 1505 CpG loci associated with 807 cancer-related genes. We previously determined that this array showed high reproducibility; results obtained in FFPE tissues were highly correlated with those from matched non-FFPE samples (*r* =0.97), and published tumor-specific methylation profiles were detectable when DNA specimens contained at least 70% tumor cells [21].

Bead arrays were run in the Mammalian Genotyping Core laboratory at UNC at Chapel Hill. The Illumina GoldenGate methylation assay was performed as described previously [22] and imaged by using the BeadArray Scanner. Methylation status of the interrogated CpG sites was determined by comparing the ratio of the fluorescent signal from the methylated allele with the sum from the fluorescent signals of both methylated and unmethylated alleles. Controls for methylation status used on each bead array included the Zymo Universal Methylated DNA Standard as the positive, fully-methylated control, and a GenomePlex (Sigma-Aldrich, St. Louis, MO, USA) whole genome amplified DNA used as the negative, unmethylated control. Array data have been deposited in Gene Expression Omnibus under accession number GSE51557.

### Array data filtering and quality control

Data were assembled by using GenomeStudio Methylation software from Illumina (San Diego, CA, USA). All array data points were represented by fluorescent signals from both methylated (Cy5) and unmethylated (Cy3) alleles. The methylation level of individual interrogated CpG sites was represented by the β value, defined as the ratio of fluorescent signal from the methylated allele to the sum of the fluorescent signals of both the methylated and unmethylated alleles and calculated as β = max(Cy5,0)/(|Cy5| + |Cy3| +100) [22]. β values ranged from 0 in the case of completely unmethylated to 1 in the case of fully methylated DNA.

Methylation array profiling was initially performed on 625 primary breast tumors by using the Illumina Cancer Panel I array that contained a total of 1,505 CpG probes. A series of filtering steps were then carried out as follows: (1) tumors with more than 25% unreliable detection *P* values of more than 10^-5^ were removed (n =14) [23]; (2) 411 CpG probes that were previously reported to overlap a single-nucleotide polymorphism (SNP) or repeat [24] were removed since these probes were potentially unreliable in some samples, especially in a racially diverse dataset such as CBCS; (3) CpG probes were removed with detection *P* value of more than 10^-5^ (n =19); (4) CpG probes with standard deviation of less than 0.06 (n =140) were removed according to Illumina's quality control algorithm [25]. Three tumors were removed because they became ineligible for the study. Finally, data from 90 tumors with replicate samples (89 with duplicates and 1 tumor with triplicates) were averaged. β values of replicate samples were highly correlated, having Pearson correlations of more than 0.900 for all but five tumors, with the remaining five tumor sets having correlations ranging from 0.899 to 0.720. The final data set consisted of 935 CpG loci (within 609 genes) in 517 breast tumors. All subsequent statistical analyses were carried out by using the R statistical programming language [26], with specific R functions noted below.

### Consensus clustering and differential methylation analysis

Consensus clustering [27] was performed by using ConcensusClusterPlus [28] and CalclCL functions in R to determine subgroups of tumors on the basis of the most variable CpG sites; sites with standard deviation less than 0.2 were excluded, leaving 167 sites. This algorithm determines "consensus" clusters by measuring the stability of clustering results from the application of a given clustering method to random subsets of the data. In each iteration, 80% of the tumors were sampled, and the k-means algorithm, with the Euclidean squared distance metric, was used with k =2 to k =10 groups; these results were compiled over 100 iterations, and the stability of each clustering was determined. We chose the greatest number of clusters that had at least 90% cluster consensus. The consensus cluster heatmap was constructed by using the gplots and heatmap.2 functions in R.

Non-parametric Wilcoxon rank sum (for two class) and Kruskal-Wallis (for multiclass) tests were used to identify CpG sites that were significantly differentially methylated between tumor subgroups identified by consensus clustering. Multivariate analyses were conducted by using general linear regression models fitted to the logit transformed β methylation values to assess the association between methylation at each CpG locus and clinical or tumor covariates, adjusting for age, race, menopausal status, and stage as appropriate. *P* values were adjusted by using the Benjamini-Hochberg false discovery rate (FDR) [29] to adjust for multiple comparisons, and probes were selected at FDR of 0.05. Volcano plots were used to display global association patterns of differential methylation in which the estimated coefficients from multivariate analysis for grade, tumor size, or clinical stage were plotted against the negative logarithm of the *P* values obtained from the hypothesis test if the estimated coefficients are non-zero. The volcano plots were plotted by using raw *P* values (that is, not adjusted for multiple comparisons).

### Survival analyses

Kaplan-Meier plots were used to illustrate disease-specific or overall survival among breast tumor clusters defined by methylation profiles. Survival analyses were carried out by using the survival package in R [30]. To identify methylation profiles associated with survival, multivariate Cox proportional hazard models [31] were fit with methylation cluster indicator by using R functions coxph [32] and cox.zph [33], with demographic and clinical attributes (age, race, menopausal status, stage, and other prognostic factors) as covariates. The *P* values for the Cox regression coefficients were adjusted by using Benjamini-Hochberg FDR for multiple comparisons [29].

### Gene ontology term enrichment analysis for groups of differentially methylated genes

The DAVID (Database for Annotation, Visualization and Integrated Discovery) Bioinformatics Resources 6.7 Functional Annotation Tool [34] was used to perform gene-gene ontology (GO) term enrichment analysis to identify the most relevant GO terms associated with the genes found to be differentially methylated between breast tumor subsets defined by intrinsic subtype, hormone receptor (HR) status, p53 mutation status, or methylation cluster (for example, the hypermethylation cluster 3 versus other clusters). DAVID calculates an enrichment score and enrichment *P* value for each GO term to highlight the most relevant GO terms associated with the selected gene list. We used the Entrez gene IDs from each list and compared these with the background list of 609 genes evaluated from the Illumina Cancer Panel I array after filtering. Genes with more than one CpG site were listed only once in the analysis. We performed functional annotation clustering with default settings. Terms that were significantly enriched (FDR *P* <0.05) are listed.

### Validation using The Cancer Genome Atlas data

Breast tumor methylation and gene expression data from 581 breast cancer patients in The Cancer Genome Atlas (TCGA) [35] were used to validate and test for relationships with gene expression at CpG probes that were among the top differentially methylated markers in the CBCS. TCGA breast cancer patients were older than CBCS patients (69% >50 years compared with 36% in CBCS), included few blacks (9% compared with 42% in CBCS), and had more later-stage 3 or 4 disease (26% compared with 12% in CBCS). Only 371 CpG probes from the GoldenGate array exactly matched probes on the Illumina 450 K array used in TCGA. Of the 935 CpG probes interrogated on the Illumina GoldenGate platform in CBCS, 21 were among our top differentially methylated probes and had exact matches for probes on the 450 K array. Based on these 21 matched 450 K CpG probes, correlations were determined with gene expression by using RNAseq expression data for all tumors (n =581) and separately for basal-like (n =102) and luminal A (n =321) tumors classified by PAM50. Pearson correlation coefficients were calculated on the basis of RNAseq (Illumina) log2 RSEM gene-normalized expression values with methylation β values for 450 K CpG probes, with significance set at *P* value of less than 0.05.

## Results

### Characteristics of breast cancer cases evaluated for promoter methylation

Demographic and clinical characteristics of the 517 breast cancer cases whose tumors were evaluated for Illumina promoter methylation are detailed in Table 1. The mean age of cases was 49.7 years, with 62% age 50 or younger, and 53% premenopausal. Breast cancer cases were mostly early-stage (88% stages 1 or 2), node-negative (58%), and HR^+^ (ER^+^ or PR^+^ or both) (68%). Intrinsic tumor subtypes, defined by a panel of IHC markers (ER, PR, HER2, CK5, CK6, EGFR), identified 51% luminal A, 16% luminal B, 21% basal-like, 6% HER2^+^/HR^-^, and 6% unclassified. Histologic subtypes included approximately 75% ductal and 18% lobular or mixed lobular histologic types. Nearly all tumors were rigorously screened for p53 gene mutations, and 42% were mutation-positive. Compared with cases not evaluated (n =163), cases who were methylation profiled were younger (p =0.02) and were marginally more likely to have p53 mutation-positive tumors (*P* =0.08).

### Consensus clustering of methylation β values of the most variant CpG loci in breast tumors identifies distinct molecular and subtype-related signatures

**Figure 1:**
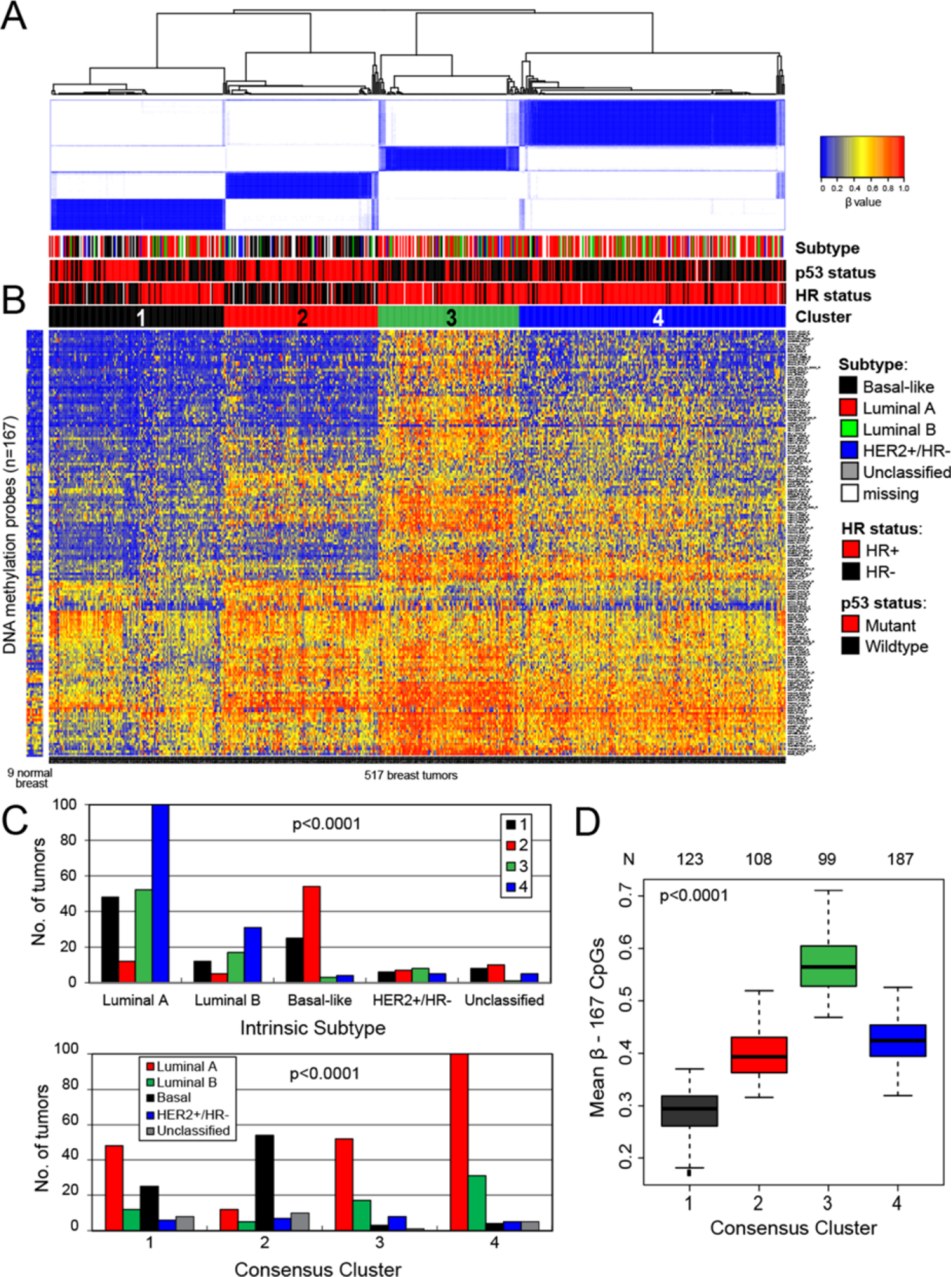
**Consensus clustering of methylation β values in breast tumors using the GoldenGate Cancer Panel I array.** DNA methylation profiles in 517 breast tumors and 9 normal breast tissues are shown. Columns represent tissue samples; rows represent CpG (cytosine preceding a guanosine) loci. Beta (β) value, indicating the fraction of DNA methylated, varies from 0 (blue, unmethylated) to 1 (red, highly methylated), with intermediate values shown in yellow. **(A)** Unsupervised clustering of the 167 most variable CpG sites having standard deviation of methylation β values of more than 0.2 from among the 935 CpG sites evaluated after filtering (see Materials and methods). The four tumor clusters are numbered 1 (n =123), 2 (n =108), 3 (n =99), and 4 (n =187). Primary tumor characteristics are indicated at the top of the heatmap as intrinsic subtype (defined by immunohistochemistry, or IHC): luminal A (red), luminal B (green), basal-like (black), HER2^+^/ER^-^ (blue), unclassified (gray), missing values (white); p53 mutation status: mutant (red), wild-type (black); and hormone receptor (HR) status: HR^+^ (red), HR^-^ (black). **(B)** Methylation in the same 167 CpG sites in 9 normal breast tissues, with probes ordered as in the consensus clustered heatmap. **(C)** Relationship between methylation cluster and intrinsic tumor subtype, shown according to intrinsic subtype (top panel) or according to methylation cluster (bottom panel). **(D)** Box-and-whisker plot showing differences (*P* <0.0001) in methylation (β) of the four consensus clusters, with numbers of tumors within each cluster shown along the top of the boxplot. Luminal-enriched tumor cluster 3 exhibits distinctly higher methylation than other clusters. ER, estrogen receptor; HER2, human epidermal growth factor receptor 2.

In total, 935 CpG probes (listed in Additional file 2: Table S1) were successfully screened for methylation in 517 primary breast tumors; because initial clustering indicated that many of these exhibited negligible variation in methylation level across tumors in the dataset, we performed consensus clustering by using the most variant 167 CpG probes (with standard deviation >0.2) (listed in Additional file 3: Table S2) in order to focus on CpG sites that were more likely to be useful for the subclassification of tumors. As illustrated in Figure 1A, four distinct clusters of breast tumors (numbered 1 to 4) were determined by consensus clustering when using 90% cluster consensus across all clusters as the criterion. Methylation profiles for nine normal breast tissues obtained from healthy/non-cancer patients from the UNC Tissue Procurement core are shown as a separate panel in Figure 1B, with probes similarly ordered. For the most variant probes, mean β values for each consensus cluster, together with those for normal breast tissues, are provided in Additional file 4: Table S3.

The four methylation-defined tumor clusters differed in their demographic, clinical, and molecular characteristics (Figure 1A and C and Table 2). In particular, cluster 2 was highly enriched for HR^-^ tumors (80%) and contained the highest proportion of basal-like tumors (61%). Fewer basal-like tumors were found in clusters 1 (25%), 3 (4%), and 4 (3%). As expected, basal-enriched cluster 2 contained tumors that were of higher grade, larger size (>2 cm), stage 2 or higher, and more frequently p53 mutant. In contrast, clusters 3 and 4 were highly enriched for HR^+^ luminal breast tumors, containing mainly mixtures of luminal A (HER2^-^) and luminal B (HER2^+^) subtypes. Cluster 4 contained the largest proportion of luminal tumors (91%), followed by clusters 3 (85%), 1 (61%), and 2 (20%). Luminal-enriched clusters 3 and 4 exhibited fewer p53 mutations compared with the basal-enriched cluster 2. Moreover, although cluster 3 consisted of mostly luminal breast cancers, these cases exhibited the highest lymph node positivity (50%) of the four tumor clusters and larger tumor size and higher stage (>stage 2) similar to tumors in basal-enriched cluster 2. Differences in age (*P* =0.006) and race (*P* =0.02) were noted among the four clusters, with the cases in basal-enriched cluster 2 being somewhat younger (<50 years) and more frequently African-American compared with the other clusters.

**Table 2:** **Characteristics of the four methylation-based consensus clusters**

Characteristic	Cluster 1		Cluster 2		Cluster 3		Cluster 4		*P*value
	(n =123)		(n =108)		(n =99)		(n =187)		
	N	(%)	N	(%)	N	(%)	N	(%)	
Age, years									
<50	73	(59)	82	(76)	54	(55)	109	(58)	0.006
50+	50	(41)	26	(24)	45	(45)	78	(42)	
Race									
White/Other	66	(54)	53	(49)	58	(59)	124	(66)	0.02
African-American	57	(46)	55	(51)	41	(41)	63	(34)	
Menopausal status									
Postmenopausal	61	(50)	39	(36)	51	(52)	91	(49)	0.09
Premenopausal	62	(50)	69	(64)	48	(48)	96	(51)	
Stage									
I	58	(49)	26	(27)	24	(25)	70	(41)	0.02
II	48	(40)	58	(60)	56	(60)	83	(48)	
III	12	(10)	9	(9)	11	(12)	13	(8)	
IV	1	(1)	4	(4)	3	(3)	5	(3)	
Missing	4		11		5		16		
Primary tumor size									
≤2 cm	72	(60)	35	(35)	36	(38)	107	(59)	0.0003
>2-5 cm	39	(33)	53	(54)	49	(51)	64	(35)	
>5 cm	9	(7)	11	(11)	11	(11)	11	(6)	
Missing	3		9		3		5		
Lymph node status									
Positive	44	(37)	40	(39)	48	(50)	75	(42)	0.24
Negative	76	(63)	62	(61)	48	(50)	105	(58)	
Missing	3		6		3		7		
Tumor grade									
I	29	(24)	3	(3)	17	(17)	77	(42)	<0.0001
II	34	(28)	16	(15)	40	(40)	66	(36)	
III	58	(48)	87	(82)	42	(43)	41	(22)	
Missing	2		2				3		
Estrogen receptor status									
Positive	56	(47)	11	(11)	79	(81)	153	(83)	<0.0001
Negative	64	(53)	90	(89)	18	(19)	31	(17)	
Missing	3		7		2		3		
Hormone receptor status									
Positive	69	(58)	20	(20)	83	(86)	166	(90)	<0.0001
Negative	51	(42)	81	(80)	13	(14)	18	(10)	
Missing	3		7		3		3		
Histologic type									
Ductal NOS	98	(80)	89	(82)	78	(79)	123	(66)	<0.0001
Ductal variants	3	(2)	0	(0)	4	(4)	6	(3)	
Poorly differentiated	7	(6)	11	(10)	2	(2)	2	(1)	
Lobular	8	(6)	1	(1)	7	(7)	30	(16)	
Mixed lobular	7	(6)	7	(7)	8	(8)	26	(14)	
Intrinsic subtype (IHC)									
Luminal A	48	(49)	12	(14)	52	(64)	100	(69)	<0.0001
Luminal B	12	(12)	5	(6)	17	(21)	31	(22)	
Basal-like	25	(25)	54	(61)	3	(4)	4	(3)	
HER2^+^/ER^-^	6	(6)	7	(8)	8	(10)	5	(3)	
Unclassified	8	(8)	10	(11)	1	(1)	5	(3)	
Missing	24		20		18		42		
p53 mutation status									
Mutant	59	(48)	90	(83)	25	(25)	44	(24)	<0.0001
Wild-type	64	(52)	18	(17)	74	(75)	141	(76)	
Missing							2		
EGFR status									
Positive	34	(32)	75	(75)	9	(10)	11	(7)	<0.0001
Negative	71	(68)	25	(25)	78	(90)	149	(93)	
Missing	18		8		12		27		
HER2 status									
Positive	25	(20)	16	(15)	31	(31)	50	(27)	0.02
Negative	97	(80)	92	(85)	68	(69)	135	(73)	
Missing	1						2		

Hormone receptor (HR) status: positive: estrogen receptor-positive (ER^+^) or progesterone receptor-positive (PR^+^) or both; negative: ER^-^ and PR^-^. Consensus methylation clusters 1 to 4 based on the most variant 167 CpG (cytosine preceding a guanosine) sites. Intrinsic subtypes: luminal A (ER^+^ and/or PR^+^, HER2^-^), luminal B (ER^+^ and/or PR^+^, HER2^+^), basal-like (ER^-^, PR^-^, HER2^-^, CK5^+^ and/or CK6^+^ or EGFR^+^), HER2^+^/HR^-^ (ER^-^/PR^-^/HER2^+^), and unclassified (all markers negative). EGFR, epidermal growth factor receptor; HER2, human epidermal growth factor receptor 2; IHC, immunohistochemistry; NOS, not otherwise specified.

The average methylation content of each cluster, estimated by using the mean methylation (β) values across the 167 most variant CpG sites, differed significantly between consensus clusters (*P* <0.0001), with the luminal-enriched cluster 3 exhibiting the highest mean β overall, cluster 1 containing the lowest level of methylation, and basal-enriched cluster 2 and the highly luminal-enriched cluster 4 exhibiting intermediate levels (Figure 1D).

### Cluster hypermethylation gene signature

To identify the hypermethylated CpG loci that defined luminal-enriched cluster 3, the Wilcoxon rank sum test was used to compare mean β at each of the 167 CpG sites in cluster 3 versus all other breast tumors. Cluster 3 showed significant differential methylation at 149 CpGs in 116 genes compared with all other breast tumors after accounting for multiple comparisons (Additional file 5: Table S4 and Additional file 6: Figure S1); the great majority of loci, though not all, were relatively hypermethylated in cluster 3 to varying degrees, with such genes as *ASCL2*, *GFI1*, *IPF1* (or *PDX1*), *IRAK3*, *ISL1*, *JAK3*, *KIT*, *MME*, *PENK*, *RARA*, *RASSF1*, *SEPT9*, *VIM*, and *WT1* showing the largest differential methylation. Of the 149 cluster 3-defining CpGs, 92 were unmethylated or poorly methylated (mean β <0.2) in normal breast tissues. The cluster 3-defining gene set was enriched in homeobox genes and other developmental transcription factors: *HOXB13*, *HOXA5*, *HOXA9*, *ISL1*, *EYA4*, *ASCL2*, *IHH*, *IPF1*, *ONECUT2*, *PAX6*, *SOX1*, *SOX17*, *TBX1*, and *GAS7*. To assess the functions of the 116 genes in the cluster 3 signature, a GO search performed via DAVID Bioinformatics Resources 6.7 identified 49 significant terms (FDR *P* <0.05) related to various aspects of cellular, tissue, and organ development; cell differentiation; hormonal response; cell communication; and cell motility (Additional file 7: Table S5). Additionally, the CBCS cluster 3 hypermethylation signature showed substantial overlap with the `methyl-deviator' signature at the CpG probe (n =64) or gene (n =60) level described in the study of Killian *et al.*[36] that also used the Illumina Cancer Panel I array (Additional file 5: Table S4 and Additional file 8: Figure S2A). Comparing the significant hypermethylated probes from CBCS cluster 3 with those from the `methyl deviator' signature, each identified from the 1505 CpG-probe GoldenGate background, we observed a highly significant correlation (*P* <0.0001, Fisher's exact test), even though different algorithms were employed to derive each of these signatures. Moreover, despite the enormous difference in the numbers of CpGs interrogated between the Illumina GoldenGate and 450 K array platforms used in the CBCS versus TCGA, respectively, genes in our hypermethylated cluster 3 were also found within the hypermethylated tumor cluster reported in TCGA (Additional file 5: Table S4) [35].

### Identification of genes differentially methylated according to clinicopathologic characteristics

**Figure 2:**
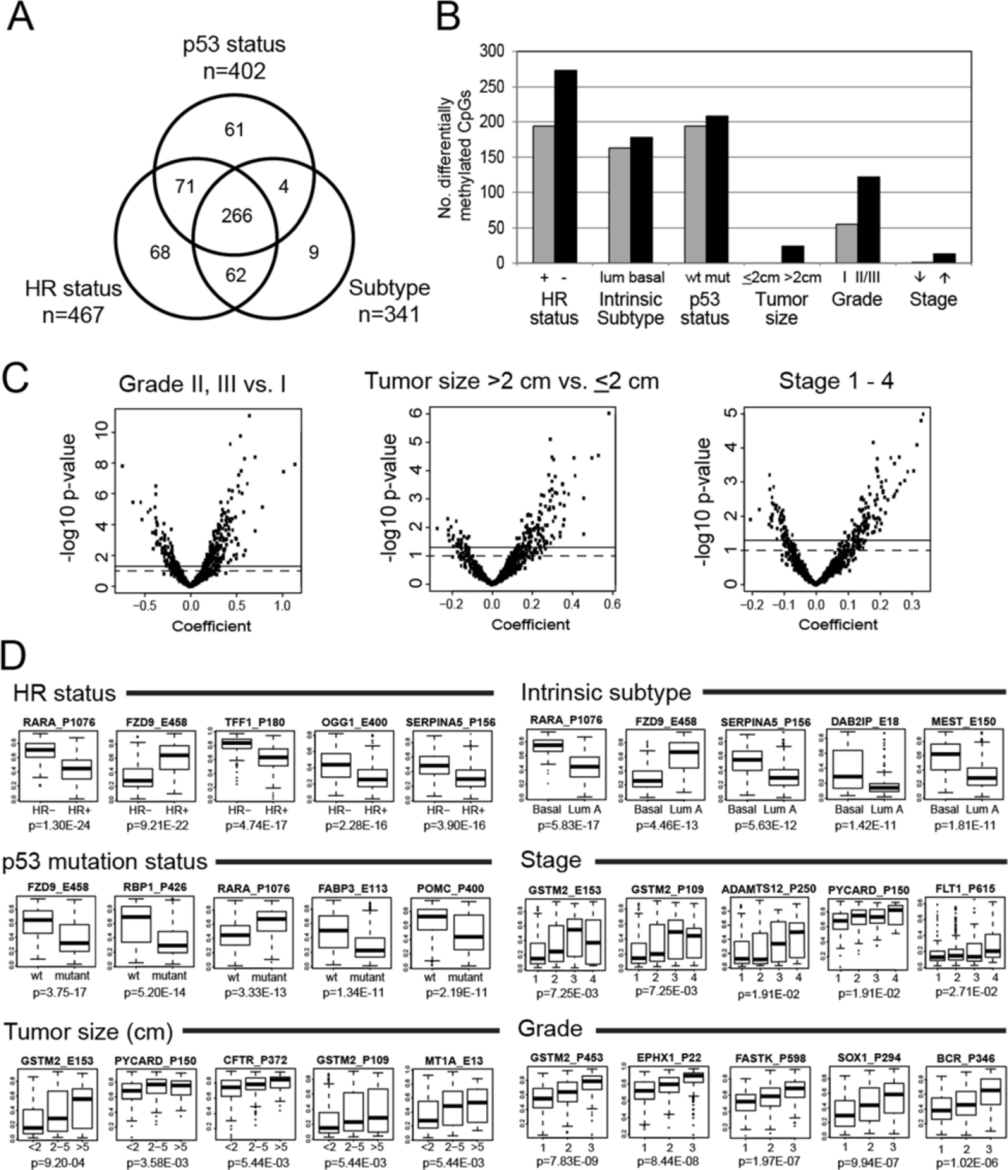
**Differential CpG methylation in breast tumors according to clinical or tumor factors.** Generalized linear regression models were used to compare methylation at each of 935 CpG sites in breast tumors according to clinical or prognostic factors while controlling for age, race, menopausal status, and stage (except in analyses of tumor size or nodal status; tumor size was adjusted for in the analysis of nodal status, and vice versa). **(A)** Venn diagram showing overlap of significantly differentially methylated sites (false discovery rate (FDR) *P* <0.05) according to hormone receptor (HR) status, intrinsic subtype (basal-like versus luminal A), and p53 status. Full lists of differentially methylated CpG loci are given in Additional files 9, 10, and 11: Tables S6-S8. **(B)** Bar graph summarizing the numbers of differentially methylated CpG loci that were relatively hypermethylated or hypomethylated in association with clinical or tumor characteristics. For analysis of stage, methylation varied between stages 1 to 4. **(C)** Volcano plots showing global patterns of differential methylation across all 935 CpGs. All multivariate models were adjusted for age, race, menopausal status, stage, except for stage (adjusted for age, race, and menopausal status only), and tumor size (adjusted for age, race, menopausal status, and lymph node status). Probes significantly differentially methylated at the *P* <0.05 level in multivariate analysis fall above the solid line and at *P* <0.1 above the broken line. **(D)** Box-and-whisker plots showing the top five CpGs exhibiting significant differential methylation according to clinical staging or tumor characteristics. Each box plot shows the median β-value (dark bar within box) and the interquartile range (IQR = Q3-Q1) (outer boundaries of box). The whiskers (broken line) cover (Q1 − 1.5IQR, Q3 + 1.5IQR). Multivariate and FDR-adjusted *P* values are shown for each boxplot. No CpGs were differentially methylated according to lymph node status.

In addition to unsupervised analysis, we looked for patterns that varied as a function of specific clinical characteristics across all 935 CpG loci. Multivariate linear regression analysis controlling for age, race, menopausal status, stage, and multiple comparisons using FDR identified 467 CpG sites in 350 genes that were significantly (*P* <0.05) differentially methylated according to HR status (ER^+^ or PR^+^ or both versus ER^-^/PR^-^), 341 CpG sites in 264 genes that were significantly differentially methylated between basal-like and luminal A breast tumor subtypes, and 402 CpG sites in 296 genes that were significantly differentially methylated between p53 mutant and wild-type breast tumors. Complete lists of differentially methylated CpG loci are provided in Additional files 9, 10, and 11: Tables S6-S8. After controlling for intrinsic subtype in the regression model for p53 mutation, 164 significantly differentially methylated CpGs persisted, suggesting that some p53-related methylation events are independent of subtype. There was considerable overlap in the CpG loci (n =266) differentially methylated by HR status, intrinsic subtype, and p53 status (Figure 2A), with only 68 CpGs, 9 CpGs, and 61 CpGs being uniquely differentially methylated, respectively. Similar numbers of differentially methylated CpG loci were relatively hypermethylated or hypomethylated in association with HR status, subtype, or p53 mutation status (Figure 2B). Genes more highly methylated in HR^+^, luminal, or p53 wild-type breast cancers included *FABP3*, *FGF2*, *FZD9*, *GAS7*, *HDAC9*, *HOXA11*, *MME*, *PAX6*, *POMC*, *PTGS2*, *RASSF1*, *RBP1*, and *SCGB3A1*; among the genes more highly methylated in HR^-^, basal-like, or p53 mutant tumors were *BCR*, *C4B*, *CDH17*, *DAB2IP*, *MEST*, *RARA*, *SEPT5, SERPINA5*, *TFF1*, and *THY1*. Among the p53-related genes, 34 were also associated with p53 mutation status in the study of Ronneberg *et al.*[37] (Additional file 8: Figure S2B).

Volcano plots were used to visualize patterns of differential methylation across all CpGs according to grade, tumor size, or clinical stage, showing the coefficients from multivariate analyses and associated log^-10^*P* values (Figure 2C). Higher tumor grade (II/III versus I), larger tumor size (<2 cm versus >2 cm), and increasing clinical stage (comparing across stages 1 to 4) were associated primarily with CpG hypermethylation; however, compared with HR status, subtype, or p53 status, fewer differentially methylated CpG loci were detected in association with these characteristics (177 CpGs for tumor grade, 24 CpGs for tumor size, and 14 for stage) (Figure 2B). Higher methylation at one or more CpG sites in the upstream regulatory region of *GSTM2* was correlated with increasing stage, larger tumor size, and higher grade. Similarly, higher methylation in the *MT1A* gene was correlated with larger tumor size. No CpG probes were found to be differentially methylated in relation to lymph node status. Boxplots showing the distribution of β values for the top differentially methylated CpG loci (at FDR *P* <0.05 level) in breast tumors according to clinicopathologic factors are given in Figure 2D. We also tested for differences in tumor methylation with age among premenopausal or postmenopausal cases, but no significant differences were detected after adjustment for multiple comparisons.

GO analysis of 350 genes that were differentially methylated between HR^+^ and HR^-^ breast tumors identified 71 GO terms that remained highly significant after adjusting for multiple comparisons (FDR adjusted *P* <0.05); similarly, GO analysis for 264 genes differentially methylated between luminal A and basal tumor subtypes identified 36 terms, and analysis of 296 genes differentially methylated between p53 mutant and wild-type tumors identified 44 GO terms. As expected, there was considerable overlap in the terms identified in these three analyses (Additional file 12: Table S9), which were related to signal transduction, anatomic development, cell differentiation and cell proliferation, and response to steroid hormone stimulus. Additional GO terms related to HR status included regulation of cell death, apoptosis, and programmed cell death.

### Survival differences among methylation-based consensus clusters

**Figure 3:**
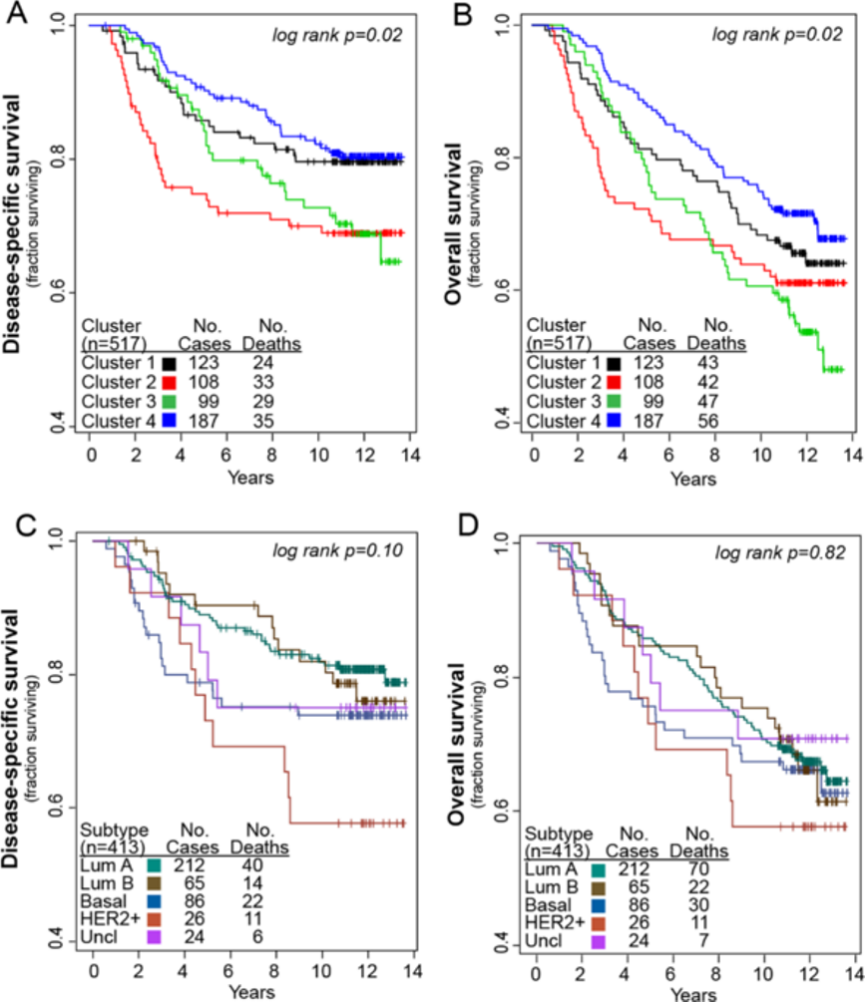
**Kaplan-Meier plot showing survival of breast cancer case subsets defined by methylation-based consensus clustering or intrinsic subtyping.** Consensus clustering of methylation β values for the 167 most variant CpG (cytosine preceding a guanosine) sites in 517 breast tumors defined four clusters. Kaplan-Meier plots show **(A)** breast cancer-specific survival or **(B)** overall survival for methylation-based clusters 1 through 4. Log-rank *P* values (*P* =0.02) indicate significant differences in survival among the methylation clusters. Intrinsic subtype information was available on 413 of the 517 tumors with methylation data. Subtypes defined by immunohistochemistry (IHC), as described in the Materials and methods section, were luminal A, luminal B, basal-like, human epidermal growth factor receptor 2-positive/hormone receptor-negative (HER2^+^/HR^-^), and unclassified. Kaplan-Meier plots for the five intrinsic subtypes show **(C)** breast cancer-specific survival and **(D)** overall survival. Numbers of cases and events within each group are noted in each plot.

Kaplan-Meier curves showing breast cancer-specific survival of CBCS cases revealed some differences between the four tumor subgroups defined by methylation signature (log-rank *P* =0.02) (Figure 3A). The luminal-enriched hypermethylated cluster 3 and the basal-enriched cluster 2 showed poorer survival compared with clusters 1 and 4. Although basal-enriched cluster 2 showed worse early survival, cluster 3 showed similar survival to the basal-enriched cluster by the end of the follow-up period. Overall survival was worse than breast cancer-specific survival for all clusters but generally reflected the relative differences noted between methylation-based clusters, with hypermethylated cluster 3 showing somewhat worse long-term survival than the other three clusters (Figure 3B). Figures 3C and D show survival plots for disease-specific and overall survival based among breast tumors distinguished by intrinsic subtype. Compared with the intrinsic subtypes, methylation-based clustering provided somewhat better distinction of patients differing in outcome based on log-rank *P* values.

To determine whether methylation profile would provide superior prognostic value for breast cancer compared with the known clinical or prognostic factors, each clinical attribute as well as methylation-based tumor cluster was tested in univariate Cox proportional hazard analysis; factors identified as significant in univariate analysis were then included in multivariate models to determine whether methylation signature was an independent predictor of survival (Table 3). In univariate analyses for patient or clinical variables, age, race, menopausal status, stage, HR status, tumor size, lymph node status, and tumor grade all showed significant hazard ratios (at *P* <0.05). Relative to luminal-enriched cluster 4 reference group, basal-enriched cluster 2 and hypermethylated cluster 3 showed significantly worse outcome in univariate analyses (hazard ratio =1.91, 95% confidence interval (CI) = 1.19 to 3.08 and hazard ratio = 1.71, 95% CI = 1.04 to 2.79, respectively) but were not independently associated with survival in multivariate Cox proportional hazard models fully adjusted for all significant covariates (age, race, stage, HR status, grade, tumor size, and lymph node status). Univariate Cox proportional hazards analysis of intrinsic subtypes identified only the HER2^+^/HR^-^ subtype as differing in breast cancer-specific survival compared with the luminal A subtype (hazard ratio =2.41, 95% CI = 1.24 to 4.70), but there were no significant differences among subtypes in multivariate analyses after controlling for other clinical or tumor characteristics.

**Table 3:** **Cox multivariate regression analysis for breast cancer survival according to CpG methylation profile, clinical factors, or intrinsic subtypes**

Prognostic variable	Univariate			Multivariate^a^		
	Hazard ratio	95% CI	*P*value	Hazard ratio	95% CI	*P*value
**Methylation cluster (n =517)**						
4 (luminal-enriched) (n =187) (reference)	1.00	-		1.00	-	
1 (mixed) (n =123)	1.11	0.66-1.86	0.70	1.09	0.61-1.95	0.78
2 (basal-enriched) (n =108)	1.91	1.19-3.08	0.0075	1.41	0.76-2.64	0.28
3 (luminal-enriched) (n =99)	1.71	1.04-2.79	0.033	1.27	0.75-2.17	0.37
**Clinical factor (n =517)**						
Age at diagnosis (continuous)	0.97	0.96-0.99	0.004	0.99	0.96-1.01	0.38
Premenopausal (versus post)	1.76	1.20-2.57	0.004	1.27	0.72-2.22	0.41
African-American (versus white/other)	1.65	1.15-2.35	0.006	1.60	1.08-2.39	0.02
HR^-^ (versus HR^+^)	1.58	1.09-2.30	0.02	1.07	0.65-1.76	0.80
HER2^+^ (versus HER2^-^)	1.32	0.89-1.96	0.16	-	-	-
Stage (1, 2, 3, 4)	2.76	2.22-3.43	<0.0001	1.74	1.22-2.49	0.002
Grade 2/3 (versus 1)	2.51	1.46-4.31	0.0009	1.21	0.66-2.22	0.54
Lymph node-positive (versus negative)	5.25	3.44-8.00	<0.0001	3.40	2.03-5.71	<0.0001
Tumor size 2-5 cm (versus ≤2 cm)	2.32	1.52-3.55	0.0001	1.24	0.77-1.99	0.38
Tumor size >5 cm (versus ≤2 cm)	5.01	2.91-8.63	<0.0001	1.44	0.73-2.83	0.28
**Intrinsic subtype (n =393)** ^**b**^						
Luminal A (n =212) (reference)	1.00	-		1.00	-	
Luminal B (n =65)	1.14	0.62-2.09	0.68	0.97	0.51-1.84	0.93
Basal-like (n =86)	1.49	0.88-2.50	0.13	1.10	0.63-1.91	0.74
HER2^+^/HR^-^ (n =26)	2.41	1.24-4.70	0.01	1.06	0.48-2.34	0.88
Unclassified (n =24)	1.34	0.57-3.17	0.50	1.19	0.49-2.87	0.70

^a^Multivariate Cox proportional hazards regression models for methylation-based clusters were adjusted for age (continuous), menopausal status (pre/post), race, stage (1, 2, 3, 4), hormone receptor (HR) status, grade (1 versus 2 + 3), lymph node status, and tumor size. Multivariate Cox proportional hazards regression models for intrinsic subtypes were adjusted for age (continuous), menopausal status (pre/post), race, stage (1, 2, 3, 4), grade (1 versus 2 + 3), lymph node status, and tumor size. ^b^The reduced number of tumors included in models for intrinsic subtypes reflects missing data for subtype or other covariates. CI, confidence interval; HER2, human epidermal growth factor receptor 2.

### Correlations between methylation and gene expression for GoldenGate-matched 450 K Illumina probes in TCGA

Because no gene expression data were available for our CBCS tumors, we sought to infer locus-specific CpG methylation correlations with gene expression in publically available TCGA breast tumor data. In total, only 371 probes from the 1,505 probes in GoldenGate array exactly match those on the 450K. Of the 935 Illumina GoldenGate probes interrogated in our study after filtering, 21 were both direct matches to the CpG probes on the 450K array and were found to be differentially methylated and of interest in our study. For these 21 matched 450K probes, Pearson correlation coefficients were calculated in all breast tumors (n = 581) and for basal-like (n = 102) and luminal A (n = 321) tumors comparing RNAseq (Illumina) log2 RSEM gene-normalized expression values with methylation β values for 450K CpG probes (Additional file 13: Table S10). For the TCGA breast samples, approximately half the probes with exact matches to the GoldenGate platform and showing differential methylation in our study exhibited significant inverse correlations with gene expression; among these are *CCND2*, *DBC1*, *FGF2*, *JAK3*, *KIT*, and *SERPINA5*.

## Discussion

In this study, we describe the results of an array-based promoter methylation analysis of 935 CpG sites in cancer-related genes in a large, population-based study of mostly early-stage breast cancer. Consensus clustering of methylation levels for the 167 most variant CpG loci in 517 tumors identified four methylation-based tumor subgroups that were associated with HR status or specific intrinsic subtypes (basal-like versus luminal A), thus confirming that intrinsic subtype may be an important determinant of some epigenetic markers. However, there are also important methylation phenotypes that are heterogeneously expressed within tumor subclass. For example, although clusters 3 and 4 were both composed of mostly luminal tumors (85% and 91%, respectively), methylation profiling distinguished cluster 3 as a hypermethylated subclass with poorer clinicopathologic characteristics (larger tumor size, higher grade, and more frequently lymph node-positive) and possibly worse outcomes.

Most HR^+^ or luminal-enriched tumor clusters exhibited higher methylation across the most variant CpGs compared with HR^-^ or basal-like tumors. Genes previously observed to differ in methylation between luminal and basal-like subtypes and also noted in this study included *RASSF1*, *FZD9*, *PTGS2*, *MME*, *HOXA9*, *PAX6*, and *SCGB3A1*, which were more highly methylated in HR^+^ and luminal tumors [37]-[39], and *CDH17*, *EPHX1*, *TFF1*, *RARA*, and *MEST*, which showed higher methylation in basal-like tumors. These methylation-based clusters also differed in the prevalence of p53 mutation, which is strongly correlated with intrinsic subtype, occurring with high prevalence among basal-like tumors in the CBCS [7]. However, even after intrinsic subtype differences were controlled for, 164 significant p53-related CpG methylation differences persisted, suggesting that at least some of these methylation events are independent of tumor subtype. Methylation also varied according to clinicopathologic characteristics, with higher tumor grade being strongly correlated with hypermethylation of such genes as *GSTM2*, *EPHX1*, and *BCR*, and larger primary tumor size correlated with hypermethylation of *GSTM2*, *PYCARD*, *MYCL2*, and *MT1A*. Methylation of several of these genes has been noted previously in breast cancer [37],[40]-[43]. Moreover, methylation was significantly inversely correlated with gene expression for several of these genes in TCGA. Importantly, our findings are consistent with prior reports of heavier methylation among HR^+^ breast tumors, less methylation in basal-like tumors [35],[38],[39],[44], and significant correlation of breast tumor DNA methylation patterns with HR subtype [36],[45], gene expression-based subtype [35],[37],[39],[44],[46],[47], or p53 mutational status [37].

Recent evidence suggests that the distinct differences in methylation observed according to intrinsic breast tumor subtype may reflect the methylation patterns of different cells of origin. Lineage-specific differentiation changes might lock tumors into certain growth programs that subsequently help to drive the tumor phenotype and clinical outcome. Kamalkaran *et al.*[46] found that methylation patterns in basal tumors are similar to breast progenitor cells but that the patterns in luminal A tumors are similar to those identified in the more differentiated CD24^+^ luminal epithelial cells. Similarly, *in vitro* work by Bloushtain-Qimron *et al.*[48] reported that CD44^+^ progenitor-like cells of normal mammary epithelium were hypomethylated compared with luminal epithelial (CD24^+^ and MUC1^+^) and myoepithelial (CD10^+^) cells and that cell type-specific methylation patterns were conserved in breast cancer subtypes. Additionally, we observed differences in methylation of several genes that mediate or are markers for epithelial-to-mesenchymal transition (EMT) (for example, *NOTCH* or *VIM*) or signaling pathways (TGFβ, WNT/β-catenin, and FGF) linked to EMT [49]. Although differential methylation of some cadherins (*CDH17* and *PCDH1*) varied by subtype, HR status, and p53 mutational state, the EMT marker, CDH1, was not among them. Recently, Cohen *et al.*[50] mapped patterns of epigenetic pathway activation in breast and other tumor types and identified a gene expression pattern of EZH2 activation in luminal breast tumors, and HDAC4 pathway activation was seen in basal breast tumors. These two distinct activated pathways were mutually exclusive, supporting the idea that fundamentally different epigenetic programs characterize these tumor subtypes.

A growing number of studies have investigated the existence and possible clinical relevance of a CIMP in breast tumors, which has been described in other tumor types, most notably colorectal cancers [14],[51]-[57]. Putative CIMP or gene hypermethylation signatures have been identified in subsets of HR^+^ breast tumors that were independently associated with poorer clinical outcomes in multivariate Cox models [36],[45],[46] or with gene expression signatures indicative of poor prognosis [58]. Conversely, Fang *et al.*[59] found CIMP to be associated with HR^+^ status, reduced metastatic potential, and better survival, suggesting the possibility that the hypermethylated CIMP signature primarily distinguished intrinsic subtypes which are known to differ in survival. The CIMP hypermethylation profile described among HR^+^ tumors appears to manifest as a coordinated hypermethylation of a set of genes highly enriched for developmental transcription factors, polycomb repressor complex 2 gene targets, as well as genes involved in EMT and Wnt signaling [36],[46],[59]. A recent report from TCGA identified a hypermethylated, HR^+^ breast tumor subset with lower Wnt-pathway gene expression and fewer PIK3CA and MAP3K1 mutations [35].

It is unclear whether CIMP-associated gene hypermethylation in breast tumors reflects the degree of lineage-specific differentiation or is a biologically distinct entity occurring through another mechanism. It has been proposed that CIMP may signify an underlying global derangement in epigenetic regulation [59], possibly mediated by overexpression of DNA methyltransferase 3b [58],[60],[61]. Moreover, it is important to note that gene hypermethylation independent of CIMP may also have prognostic value in breast tumors [45],[62]-[65]. Notably, hypermethylation signatures predicted poorer outcomes in ER^-^ breast cancers [45],[62], with one study identifying a prognostic signature highly enriched in homeobox genes [45].

In the CBCS, consensus clustering of the 167 most variant CpG loci revealed a CIMP-like hypermethylated cluster 3. Although this cluster was composed of predominantly HR^+^/luminal tumors, it was associated with poorer clinicopathologic features and possibly worse prognosis, similar to basal-like breast cancers. In fact, methylation-based clustering provided similar discrimination of prognostically different subgroups as intrinsic subtyping based on IHC. The finding that DNA methylation profiling may identify breast cancer cases with worse outcomes irrespective of subtype, together with its particular suitability for FFPE tissues, suggests that methylation analysis could be useful for breast cancer prognosis. Cluster 3 tumors exhibited hypermethylated gene signatures enriched in homeobox domain and transcription factors important in development and differentiation, consistent with prior studies [36],[37],[45],[46],[59]. In particular, the cluster 3 CpG signature was similar to the `methyl deviator' signature identified by Killian *et al.*[36] that independently predicted poor prognosis among HR^+^ tumors. Moreover, the hypermethylated breast tumor gene signature identified in TCGA [35] overlapped with our cluster 3, showing both hypermethylation and reduced expression of genes such as *ASCL2*, *CCND2*, *COL1A2*, *EPHB1*, *FABP3*, *GAS7*, *IFNGR2*, *IRAK3*, *KLK10*, *POMC*, *SCGB3A1*, *SFRP1*, *SMO*, and *VCAN* (*CSPG2*).

Our findings from CBCS suggest that methylation patterns defined by the most variant CpG loci largely reflect cell lineage, as evidenced by the distinct differences in methylation patterns between HR^+^ and HR^-^ or basal-like and luminal A breast tumors, and the extreme hypermethylation of genes important in development and differentiation in a subset of mostly HR^+^ tumors. This is consistent with the idea that aberrant methylation occurs early in cancer development [66], suggesting that these methylation events may be important in carcinogenesis and could be linked with exposures that modulate risk of tumor subtypes. Our results also suggest that certain methylation events are associated with more aggressive tumor phenotypes irrespective of subtype and have the potential to provide prognostic information, consistent with other studies [36],[37],[45],[46]. Owing to high representation of incident, early-stage breast cancer cases in the CBCS dataset (with relatively few deaths), our power to detect significant and independent survival differences may have been limited, particularly among the better-prognostic HR^+^ cases. However, our results are derived from a population-based sample and therefore represent the distribution of incident breast cancer cases. Over time, extended follow-up of CBCS cases may allow more definitive ascertainment of the relationship between CpG methylation and breast cancer survival.

Major strengths of this study include the large size and population-based nature of the CBCS, inclusion of breast tumors with relatively complete histopathologic, subtyping, and outcome data. The sample size was large, allowing well-powered analysis of methylation signatures across a diverse spectrum of breast tumors. We used a stringent approach in methylation profiling by filtering out CpG probes that overlapped repeats or known SNPs, which might have produced unreliable results. A few limitations are also noted. The CBCS collected only FFPE tumor tissues that have been stored as cut sections for nearly 20 years. The difficulty in obtaining RNA of sufficient quality for gene expression array analysis from such tissues has precluded the direct comparison of promoter methylation and gene expression. Intrinsic tumor subtypes were defined by a panel of IHC protein expression markers, which may be less accurate than subtyping based on expression of 50 or more genes [67], and therefore likely resulted in some misclassification. This misclassification is most likely to occur among luminal breast cancers; however, given that the most prominent methylation differences were between luminal and basal-like breast cancers, this misclassification is unlikely to substantially alter the conclusions of the study. The data were collected on a first-generation methylation array which oversampled genes in cancer-related pathways; however, many genes on the platform had strong coverage for the best-studied methylation sites in breast cancer research. Additionally, information on treatment and breast cancer recurrence was not available in CBCS, and thus their impact on the relationship between methylation profile and survival could not be addressed.

## Conclusions

In summary, we found evidence for a strong association of DNA methylation with HR status and breast tumor subtype as well as with p53 mutation status, which is inextricably linked to subtype. Moreover, epigenetic heterogeneity within tumor subclass is supported by identification of a hypermethylated tumor cluster enriched in developmental genes among primarily HR^+^ luminal tumors. This hypermethylated signature may be related to more aggressive tumor growth features and, potentially, outcome. These findings provide proof-of-principle that epigenetic profiles may offer important information beyond expression-based subtyping for clinically or epidemiologically meaningful breast tumor classification.

## Authors' contributions

KC conceived, designed, and implemented the study; analyzed and interpreted the data; drafted the manuscript; and obtained funding for the study. HC, PFK, RM, CB, TS-S, and C-KT analyzed the data and contributed to writing the manuscript. SE participated in study design, processed tissue samples, generated the methylation data, and contributed to analysis and interpretation of data and writing of the manuscript. RM (deceased), principal investigator of the CBCS, and MT contributed to data analysis and interpretation and provided a critical review of the manuscript. As the study pathologist, JG performed histopathologic review and scoring of tumors and their grading criteria and provided a critical review of the manuscript. All authors read and approved the final version of this manuscript.

## Additional files

## Electronic supplementary material

Additional file 1: p53 mutation screening methods.(DOCX 24 KB)

Additional file 2: Table S1.: Nine hundred thirty-five CpG sites/probes evaluated for methylation. (XLSX 25 KB)

Additional file 3: Table S2.: One hundred sixty-seven most variant CpG sites. (XLSX 13 KB)

Additional file 4: Table S3.: Mean beta values for 167 probes distinguishing four consensus clusters. (XLSX 27 KB)

Additional file 5: Table S4.: One hundred forty-nine CpG sites distinguishing hypermethylated cluster 3 from other breast tumors. (XLSX 34 KB)

Additional file 6: Figure S1.: Boxplots showing distribution of β methylation values for the top CpG markers defining the hypermethylated cluster 3. β values are shown for the four consensus clusters. CpG sites or genes that overlap the `methyl deviator' signature (β) described by Killian *et al.*[36] or the hypermethylated cluster 3 described in breast tumors profiled within The Cancer Genome Atlas (β) [35] are indicated. (PNG 157 KB)

Additional file 7: Table S5.: Gene ontology (GO) terms for genes that define the cluster 3 hypermethylation signature. (XLSX 15 KB)

Additional file 8: Figure S2.: Venn diagrams showing overlap of differentially methylated CpGs/genes between CBCS and other published studies. **(A)** Overlap of the hypermethylated signature from cluster 3 in CBCS (149 CpGs, 116 genes) with the methyl-deviator signature (109 CpGs, 85 genes) identified in the study of Killian *et al.*[36], which also used the Illumina Cancer Panel I methylation platform. **(B)** Overlap of genes differentially methylated according to p53 mutation status in CBCS (402 CpGs in 296 genes) with genes included in the p53 signature (84 genes) reported in Ronneberg *et al.*[37]. CBCS, Carolina Breast Cancer Study. (PNG 56 KB)

Additional file 9: Table S6.: Four hundred sixty-seven CpGs differentially methylated by hormone receptor (HR) status. (XLSX 64 KB)

Additional file 10: Table S7.: Three hundred forty-one CpGs differentially methylated by subtype. (XLSX 49 KB)

Additional file 11: Table S8.: Four hundred two CpGs differentially methylated by p53 status. (XLSX 57 KB)

Additional file 12: Table S9.: Gene ontology (GO) terms for genes differentially methylated according to hormone receptor (HR) status, subtype, or p53 status. (XLSX 29 KB)

Additional file 13: Table S10.: The Cancer Genome Atlas (TCGA) correlations between GoldenGate-matched 450 K Illumina probe methylation and gene expression. (XLSX 15 KB)

Below are the links to the authors’ original submitted files for images.

Authors’ original file for figure 1

Authors’ original file for figure 2

Authors’ original file for figure 3

## Abbreviations

CBCS: Carolina Breast Cancer Study
CIMP: CpG island methylator phenotype
CpG: cytosine preceding a guanosine
DAVID: Database for Annotation, Visualization and Integrated Discovery
EMT: epithelial to mesenchymal transition
ER: estrogen receptor
FDR: false discovery rate
FFPE: formalin-fixed paraffin-embedded
GO: gene ontology
HER2: human epidermal growth factor receptor 2
HR: hormone receptor
IHC: immunohistochemistry
PR: progesterone receptor
SNP: single-nucleotide polymorphism
TCGA: The Cancer Genome Atlas
