# Supplementary material for: DNA methylation profiling in the Carolina Breast Cancer Study defines cancer subclasses differing in clinicopathologic characteristics and survival
Source: Breast Cancer Res. 2014 Oct 7;16:450. doi: 10.1186/s13058-014-0450-6 (PMC4303129; doi:10.1186/s13058-014-0450-6)
Supplement: Supplementary file 1 — Additional file 1: p53 mutation screening methods.(DOCX 24 KB) [file 13058_2014_450_MOESM1_ESM.docx]

**p53 Mutation Screening Methods**

p53 mutation screening was accomplished using single strand conformational polymorphism (SSCP) analysis and direct sequencing, and subsequently by microarray using the Roche p53 AmpliChip. p53 mutations detected by either method were confirmed by sequencing.

**SSCP and direct sequencing of p53 exons 4-8**  Mutations in exons 4-8 of the p53 gene were evaluated in a consecutive series of FFPE breast tumors using a stringent screening algorithm incorporating single-strand conformational polymorphism analysis (SSCP) and manual DNA sequencing (1, 2). Briefly, PCR amplification was carried out for individual exons 5-8 to generate template for SSCP and sequencing. Due to its size, exon 4 was amplified as two overlapping segments. The sequences of all primers are shown in the table below. First PCR was carried out in a 50 µl reaction mixture containing tumor lysate DNA or 100 ng genomic DNA, 500 nM each primer, 1X Cetus buffer, 200 µM each deoxyribonucleotide and 2.5 units AmpliTaq DNA polymerase (Perkin Elmer). Amplification was performed using 1 cycle at 94o for 4 min, 55o for 1 min and 72o for 1 min., followed by 33 cycles at 94o for 1 min, 55o for 1 min and 72o for 1 min. and a final extension of 1 cycle at 94o for 1 min, and 60o for 10 min. The PCR products were analyzed by electrophoresis in 10% polyacrylamide gels, and the amplified DNA fragments visualized with ethidium bromide staining under UV irradiation.

SSCP analysis of each p53 exon 4-1, 4-2, 5, 6, 7, and 8 was performed using an aliquot of first PCR product as template. SSCP of each exon was conducted at both 25^o^C and 4oC to increase the sensitivity of mutation detection. 20 µl SSCP-PCR reactions consisted of 500 nM inner primers, 1X Cetus buffer, 200 uM each dNTP, 2.5 units AmpliTaq polymerase and 100 µCi/ml 32P-α-dCTP. Cycle conditions for p53 were as described for first PCR. To obtain better resolution of exon 4-2 bands on SSCP gels, the SSCP-PCR products were digested with 5 units Alu I (Boehringer Mannheim) in 25 µl of 1X Alu I buffer. SSCP-PCR products were diluted 30 to 50-fold with 0.1% SDS/10 mM EDTA, the diluted product (3 µl) was mixed with an equal volume of stop buffer (95% formamide, 20 mM EDTA, 0.05% bromophenol blue, 0.05 % xylene cyanol FF) and denatured at 94oC for 5 min. SSCP products were subjected to electrophoresis at room temperature and at 4oC in 6% polyacrylamide non-denaturing gels (6% polyacrylamide, 1X TBE and 10% glycerol for room temperature and no glycerol for 4o). Negative controls containing only wild type p53 were included on each gel.

Samples exhibiting abnormal band migration at either temperature were sequenced in both the forward and reverse DNA strands. Mutations were independently confirmed by sequencing of a second, separately-amplified PCR product to rule out artifactual mutations. Additionally, 50 randomly selected samples that exhibited no abnormalities on SSCP were sequenced, and in no case were mutations detected.

Tumors showing altered SSCP bands were sequenced as follows. The first PCR product or gel-purified abnormal SSCP bands was used as template for asymmetric PCR to generate single forward and reverse DNA strands for sequencing. Asymmetric PCR was carried out under PCR conditions previously described but with inner primer concentrations of either 500 nM forward primer with 10 nM reverse primer or 10 nM forward primer with 500 nM reverse primer. The asymmetric PCR products were purified through Centricon-100 concentrators (Amicon). Both forward and reverse asymmetric PCR products were sequenced using a Sequenase version 2.0 sequencing kit (Amersham). After electrophoresis on 8% polyacrylamide sequencing gels and drying, the gels were exposed to X-ray films overnight. Samples were considered to be mutation-positive only if both strands exhibited the same mutation. Mutations were further verified by repeating the sequencing analysis on a second separately-amplified DNA aliquot from suspected positive samples; only those samples showing the same mutation in both PCR aliquots were considered true positives.

**p53 AmpliChip analysis of p53 exons 2-11** The p53 AmpliChip research test (Roche Molecular Systems, Pleasanton, CA) was also used to determine p53 mutation status from 100 ng of DNA extracted from FFPE tumor tissues according to the manufacturer’s protocol. The AmpliChip p53 assay was designed to detect single base pair substitutions and deletions in the entire coding region of the TP53 gene, including splice sites of exons 2–11. The AmpliChip p53 microarray consists of over 33,000 probe sets of more than 220,000 individual oligonucleotides tiled for a total of 1268 nucleotide positions of coding regions of exons 2–11. A single probe set for an interrogating base position includes five probes: one probe to hybridise to the WT, three probes to detect three possible single base-pair mutations, and one probe to detect single deletion. There are at least 24 probe sets for each nucleotide position, including both sense and anti-sense probe sequences.

A mixture-detection algorithm determined by Roche Molecular Systems was used to facilitate detection of p53 mutations in heterogeneous samples. The algorithm utilized a sample with wild-type p53 sequence as a reference, which was hybridized and scanned under identical conditions to samples with unknown p53 sequence. Mutations were detected based on the differences in hybridization intensities between the reference and unknown sample. The ability to detect differences in intensity patterns were enhanced by including redundancy at each base position. Each site on the GeneChip corresponding to a base in the p53 sequence was covered by at least two probe sets (one sense and one antisense). In addition, 300 known missense mutations in the p53 gene were also covered by an additional 14 probe sets. Possible base calls for each site included "N" (unable to make a call); "-" (single base deletion); "A"; "C"; "G"; "T" (wild-type bases); or a mixture call (e.g., "A/G", indicating the presence of a mutant base). The algorithm also assigned a score for each site containing a mutation or single base deletion. These scores were derived from the sum of mixture variables calculated from the contributing probe sets and were affected by the number of probe sets contributing to the call for a specified position. The higher the score for a given position, the greater the likelihood that site contained a mutant base. For sites containing only sense and antisense probe sets, possible scores were 1-7 for point mutations and 1-4 for single base pair deletions. For sites covered by additional probe sets, possible scores were 1-32 for point mutations and 1-10 for single base pair deletions. Only scores exceeding 13 for point mutations and 8 for single base deletions were called a mutation.

All mutations identified by the p53 AmpliChip were confirmed by manual direct sequencing of the exon carrying the mutation.

**References**

1. Millikan R, Hulka B, Thor A, Zhang Y, Edgerton S, Zhang X, Pei H, He M, Wold L, Melton LJ, Ballard D, Conway K, Liu ET. **p53 mutations in benign breast tissue**. *J Clin Oncol* 1995, **13**:2293-300.

2. Olshan AF, Weissler MC, Pei H, Conway K, Anderson S, Fried DB, Yarbrough WG. **Alterations of the p16 gene in head and neck cancer: frequency and association with p53, PRAD-1 and HPV.** *Oncogene* 1997, **14**:811-8.

3. Chiaretti S, Tavolaro S, Marinelli M, Messina M, Del Giudice I, Mauro FR et al. **Evaluation of TP53 mutations with the AmpliChip p53 Research Test in chronic lymphocytic leukemia: correlation with clinical outcome and gene expression profiling.** *Genes Chromosomes Cancer* 2011, **50**:263-74

**Oligonucleotide Primers Used for p53 SSCP and sequencing**

**_________________________________________________________________________________________________________________________________________________**

**P53 exon 5’ Primer Sequence 3’ Primer Sequence Product Size (bp)**

**_________________________________________________________________________________________________________________________________________________**

**p53 Exon 2:**

P2-5' AGCGTCTCATGCTGGATCC P2-3' GTGGCCTGCCCTTCCAATG 168

P2-5' IN CGTCTCATGCTGGATCC P2-3' IN TGGCCTGCCCTTCCAAT

**p53 Exon 3:**

P3-5' AATTCATGGGACTGACTTTCTG P3-3' AGCCCTCCAGGTCCCAGC 92

P3-5' IN CATGGGACTGACTTTCTGCT P3-3' IN TCCAGGTCCCAGCCCAAC

**p53 Exon 4 (1):**

P4(1)-5' gacctggtcctctgactgct P4(1)-3' cggtgtaggagctgctggtg 188

P4(1)-5' IN cctctgactgctcttttcac P4(1)-3' IN cggtgtaggagctgctggtg

**p53 Exon 4(2):**

P4(2)-5' tccagatgaagctcccagaa P4(2)-3' acggccaggcattgaagtct 249

P4(2)-5' IN aagctcccagaatgccagag P4(2)-3' IN tctcatggaagccagcccct

**p53 Exon 5:**

P5-5' gctgccgtgttccagttgct P5-3' ccagccctgtcgtctctcca 294

P5-5' IN ccagttgctttatctgttca P5-3' IN tgtcgtctctccagccccag

**p53 Exon 6:**

P6-5' ggcctctgattcctcactga P6-3' gccactgacaaccaccctta 199

P6-5' IN cctctgattcctacatgatt P6-3' IN accacccttaacccctcctc

**p53 Exon 7:**

P7-5' tgccacaggtctccccaagg P7-3' agtgtgcagggtggcaagtg 196

P7-5' IN gcgcagtggcctcatcttgg P7-3' IN tgtgcagggtggcaagtggc

**p53 Exon 8:**

P8-5' ccttactgcctcttgcttct P8-3' ataactgcacccttggtctc 225

P8-5' IN cctcttgcttctcttttcct P8-3' IN tctcctccaccgcttcttgt

**p53 Exon 9:**

P9-5’ gcctcagattcacttttatcacc-3' P9-3’ cattttcactgttagactggaaac 175

p9-5’in atcacctttccttgcctct-3' p9-3’in cattttgagtgttagactgg-3'

**p53 Exon 10:**

P10-5' ACTTACTTCTCCCCCTCCTC P10-3' ATGGAATCCTATGGCTTTCC 207

P10-5' IN ACTTCTCCCCCTCCTCTGT P10-3' IN GAATCCTATGGCTTTCCAAC

**p53 Exon 11:**

P11-5' CATCTCTCCTCCCTGCTTCT P11-3' CTGACGCACACCTATTGCAA 222

P11-5' IN TCCTCCCTGCTTCTGTCTCC P11-3' IN GCACACCTATTGCAAGCAAG

_________________________________________________________________________________________________________________________________________________

IN; internal primers used for SSCP, asymmetric PCR and sequencing. Exon 4 is too long for sequencing and so was split into 2 fragments for convenience. For Exon 4-1, the 3'-inner and outer primers share the same sequence.
