# Supplementary figures and images for: DNA methylation profiling in the Carolina Breast Cancer Study defines cancer subclasses differing in clinicopathologic characteristics and survival
Source: Breast Cancer Res. 2014 Oct 7;16:450. doi: 10.1186/s13058-014-0450-6 (PMC4303129; doi:10.1186/s13058-014-0450-6)

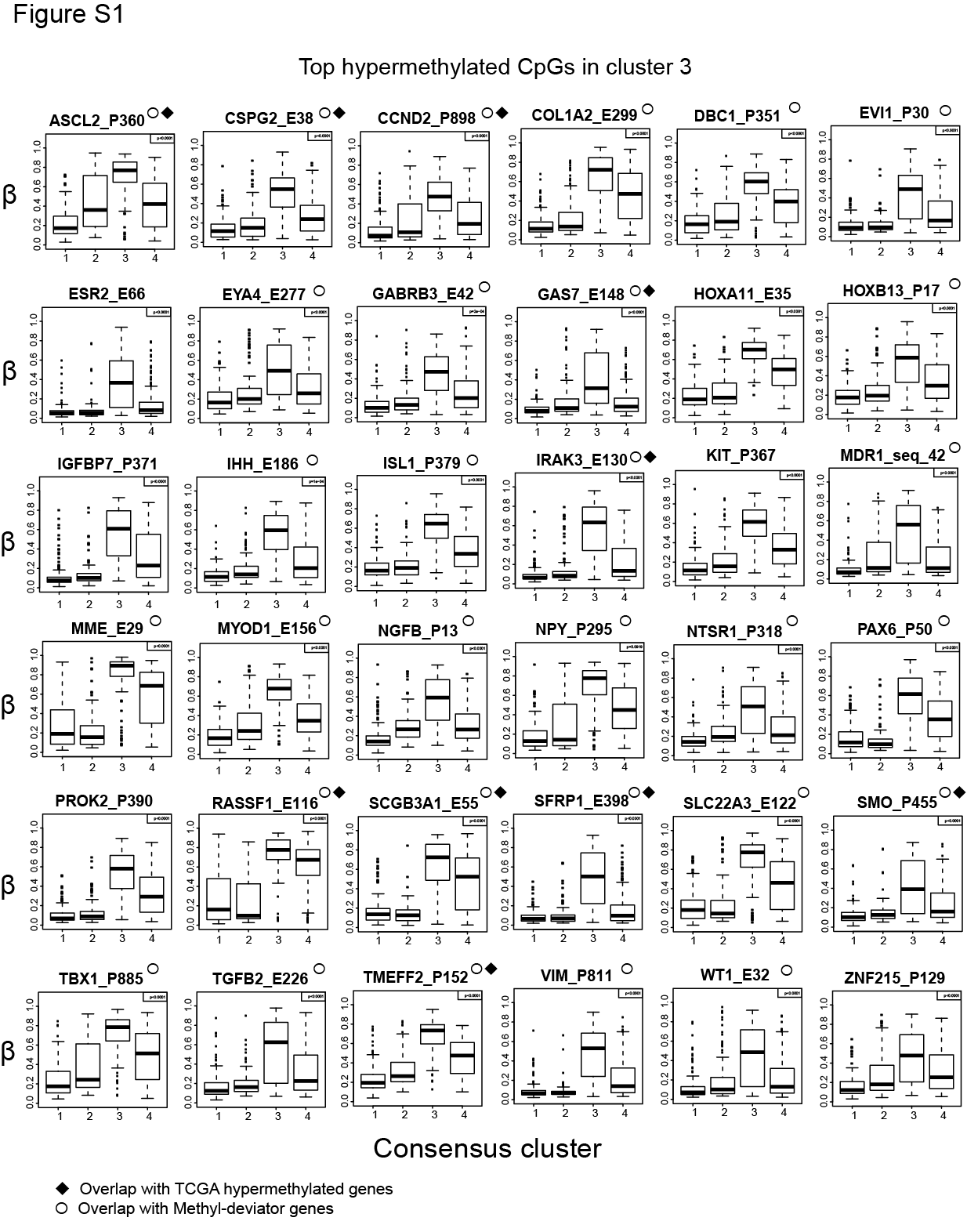

Supplement: Supplementary file 6 — Additional file 6: Figure S1.: Boxplots showing distribution of β methylation values for the top CpG markers defining the hypermethylated cluster 3. β values are shown for the four consensus clusters. CpG sites or genes that overlap the `methyl deviator' signature (β) described by Killian et al.[36] or the hypermethylated cluster 3 described in breast tumors profiled within The Cancer Genome Atlas (β) [35] are indicated. (PNG 157 KB) [file 13058_2014_450_MOESM6_ESM.png]

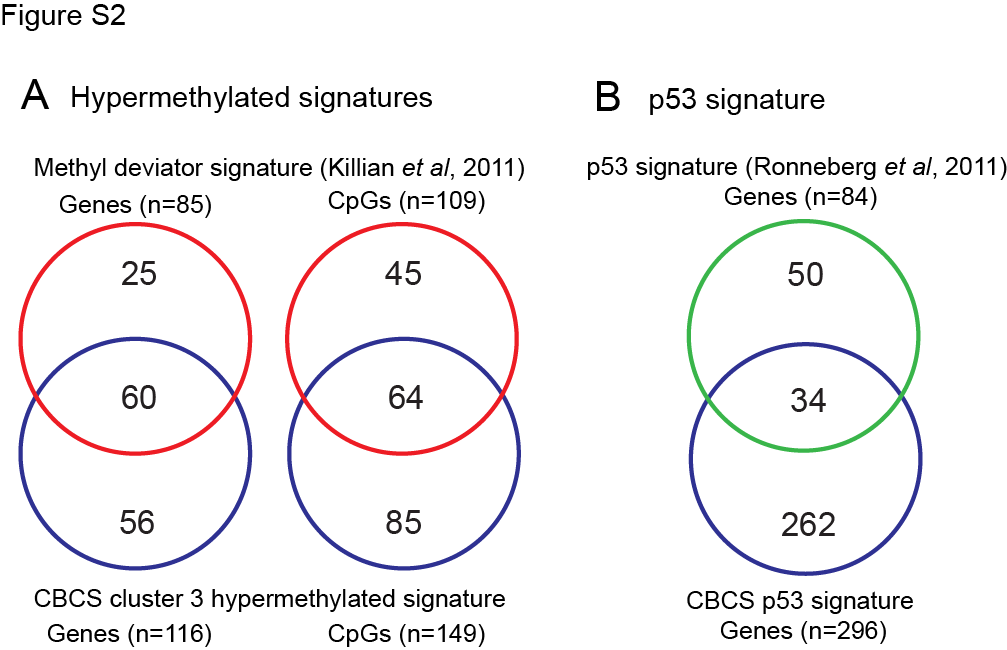

Supplement: Supplementary file 8 — Additional file 8: Figure S2.: Venn diagrams showing overlap of differentially methylated CpGs/genes between CBCS and other published studies. (A) Overlap of the hypermethylated signature from cluster 3 in CBCS (149 CpGs, 116 genes) with the methyl-deviator signature (109 CpGs, 85 genes) identified in the study of Killian et al.[36], which also used the Illumina Cancer Panel I methylation platform. (B) Overlap of genes differentially methylated according to p53 mutation status in CBCS (402 CpGs in 296 genes) with genes included in the p53 signature (84 genes) reported in Ronneberg et al.[37]. CBCS, Carolina Breast Cancer Study. (PNG 56 KB) [file 13058_2014_450_MOESM8_ESM.png]

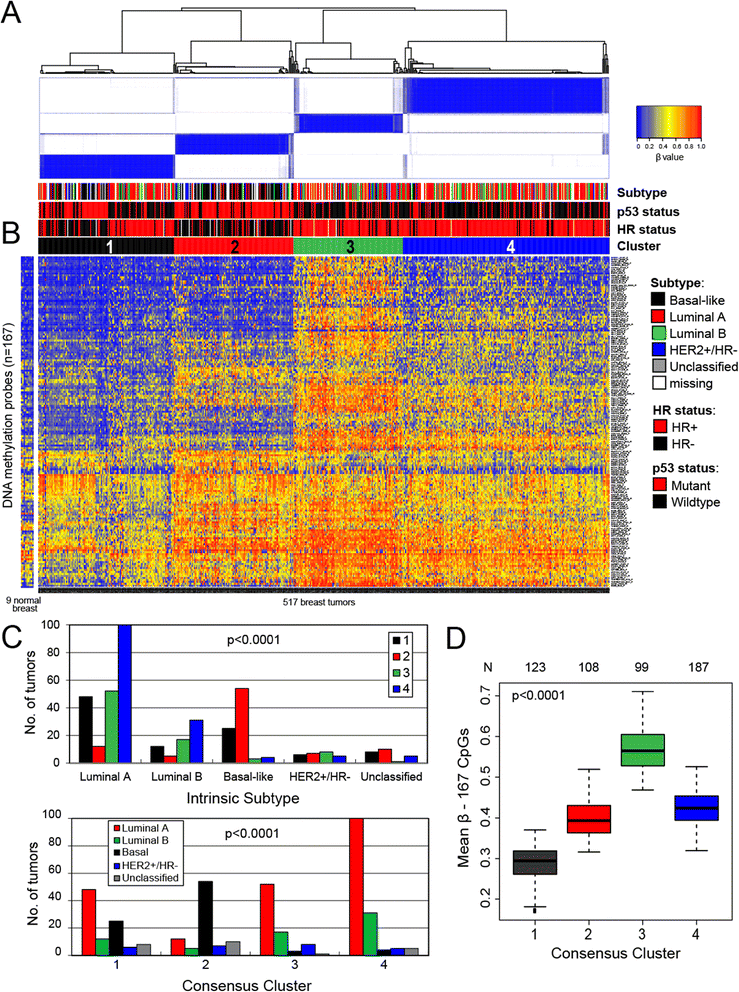

Supplement: Supplementary file 14 — Authors’ original file for figure 1 [file 13058_2014_450_MOESM14_ESM.gif]

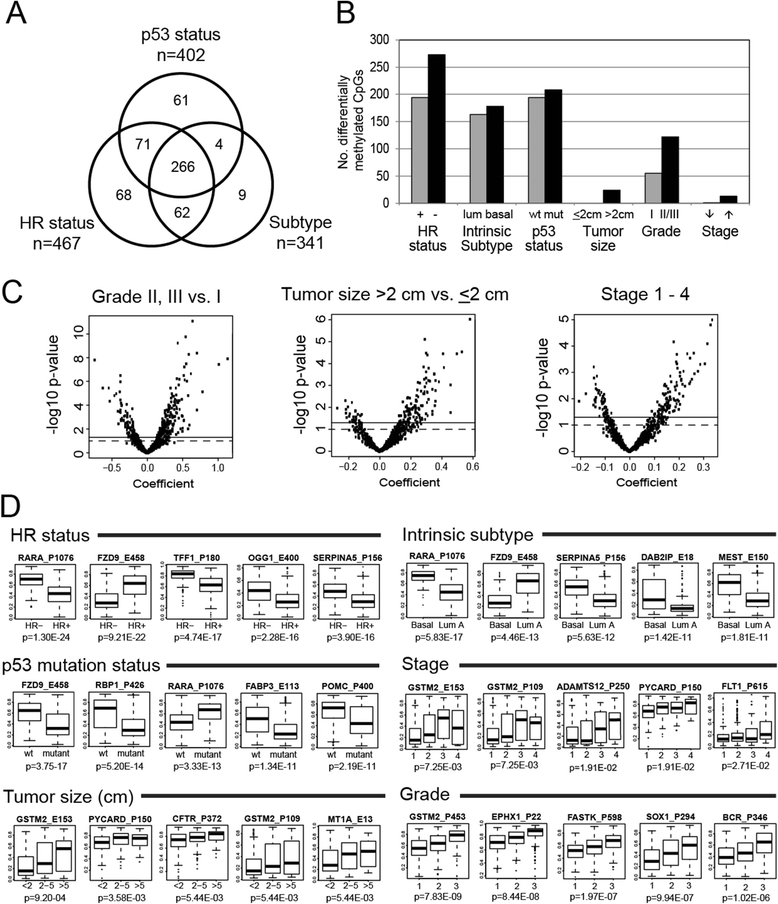

Supplement: Supplementary file 15 — Authors’ original file for figure 2 [file 13058_2014_450_MOESM15_ESM.gif]

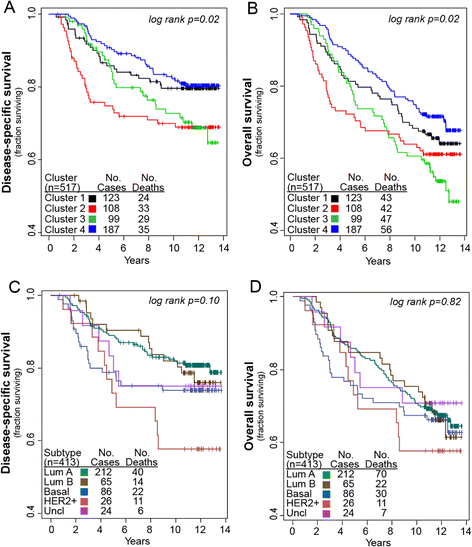

Supplement: Supplementary file 16 — Authors’ original file for figure 3 [file 13058_2014_450_MOESM16_ESM.gif]
